# Translation and validation of the cardiac rehabilitation barriers scale in the Czech Republic (CRBS-CZE): Protocol to determine the key barriers in East-Central Europe

**DOI:** 10.1097/MD.0000000000019546

**Published:** 2020-03-13

**Authors:** Petr Winnige, Ladislav Batalik, Katerina Filakova, Jakub Hnatiak, Filip Dosbaba, Sherry L. Grace

**Affiliations:** a Department of Rehabilitation, University Hospital Brno; b Department of Public Health, Faculty of Medicine, Masaryk University Brno; c Department of Internal Cardiology Medicine, University Hospital Brno, Brno, Czech Republic; d Faculty of Health, York University & University Health Network, University of Toronto, Toronto, Canada.

**Keywords:** barriers, cardiac rehabilitation, coronary artery disease, Czech Republic, enrollment, referral

## Abstract

**Background:**

Cardiovascular diseases are highly prevalent and represent leading causes of morbidity worldwide, including in Central Europe. Cardiac rehabilitation (CR) is an effective method of secondary prevention, but utilization is low. Barriers to CR use in the Czech Republic are not well-characterized, and therefore we propose a protocol to translate and validate the cardiac rehabilitation barriers scale (CRBS).

**Methods:**

In this multi-method study, we translated and cross-culturally validated the CRBS to Czech (CRBS-CZE) first through the following main steps: professional translation, reconciliation/harmonization, and cross-cultural adaptation, and piloting in 50 cardiac patients. A prospective study will be undertaken to psychometrically-validate the CRBS-CZE, where 300 to 600 cardiac inpatients eligible for phase II/outpatient CR will be recruited. Consenting participants will be informed about the CR program and their sociodemographic, clinical characteristics, and the CRBS-CZE administered. Factor analysis will be performed with oblique rotation, factors will be extracted based on eigenvalues, the examination of the scree plot, and factor loadings. The internal reliability of the total scale and subscales will be assessed with Cronbach alpha. Overall CRBS scores will be compared by patient characteristics such as sex, socioeconomic indicators, risk factor burden, and travel time to investigate content validity. Their CR enrollment, adherence (% of 24 prescribed sessions attended), and completion will be tracked. The second administration of CRBS-CZE will be undertaken in patients at 3 weeks after enrollment. To test criterion validity, *t* tests and Pearson correlation (for adherence) will be used to determine the association of these utilization indicators with CRBS scores.

**Results:**

The translated version was considered by 2 bilingual CR experts. Some revisions and example additions were made to the items. Upon piloting with patients, some further edits were made. No additional barriers were raised.

**Discussion:**

Through this study, a reliable and valid means of assessing patient's CR barriers will be established. Results will be used to identify ways to help patients overcome barriers to CR utilization.

## 1 Introduction

Cardiovascular diseases (CVDs) are the leading causes of death in the world, and are a significant burden in Central Europe in particular.^[1]^ These many cardiac patients are at increased risk for recurrent events.^[2]^ For example, CVDs are the most common reasons for hospitalization in the Czech Republic, as well as are common sick leave and disability causes.^[3]^ According to the Czech Statistical Office, 25.4 billion CZK was spent on CVDs treatment in 2016, which was 13.6% of all health care expenditures that year.^[4]^ These diseases also represent a severe socioeconomic problem for patients and their families. This is unfortunate, given, according to the World Health Organization, that nearly 75% of recurrent cardiovascular events may be prevented.^[5]^ Indeed, cardiac rehabilitation (CR) is an effective secondary preventive model of care to mitigate this burden.^[6]^ However, CR is grossly under-utilized worldwide, including in Central Europe.^[7]^

The reasons for under-utilization are well-established in high-resource settings such as Western Europe and North America, consisting of factors at the health system, provider, and patient levels.^[8–10]^ A recent review; however, reveals just how few studies have explored these barriers outside of these settings, even though the burden of CVDs is so much higher here.^[11]^

Validated scales such as the cardiac rehabilitation barriers scale (CRBS), to assess reasons for under-utilization are available in the English language.^[12]^ Despite being self-reported by patients, they can validly capture barriers at these multiple levels. Studies reveal key barriers include lack of perceived need/awareness, distance to center, and work conflicts. These are amenable to relatively simple mitigation efforts, and hence barrier identification in patients is essential to promoting their access and participation. The CRBS has now been translated into 14 languages.^[13]^ Studies in non-Western settings do reveal different barriers, barriers which again are ripe for mitigation, bolstering the importance of measuring these barriers in these settings.^[11]^

Barriers to CR participation in the Czech Republic are still unknown or not adequately described, despite this great need for CR as outlined above, as in the whole Central Europe. The aim of the study is first to translate and cross-culturally adapt CRBS to Czech (CRBS-CZE), and then psychometrically-validate the translation. The study shall include an assessment of the structure of factors, internal reliability as well as validity (design and criterion).^[14]^ The second aim then is to identify the key barriers which prevent patients from entering phase II of CR (outpatient). Furthermore, we will describe the degree of CR utilization in the program, namely, enrollment, adherence, and completion.^[15]^

## 2 Methods

### 2.1 Design and ethical considerations

This is a multi-method study, first involving a translation and cross-cultural validation process for the CRBS, and second a prospective, observational study to psychometrically-validate it. The project respects the World Medical Association Declaration of Helsinki on ethics in medical research and received approval by the Ethical Committee of the University Hospital (UH) Brno, Czech Republic. This study is registered at the Australian New Zealand Clinical Trial Registry with registration number: ACTRN12619001181190. Data will be password-protected so that only the research team can access it. Personal information will be processed and backed up according to the current General Data Protection Regulations.

### 2.2 Setting

The prevalence of cardiovascular risk factors in the Brno region is high.^[16]^ This study will be undertaken at the UH Brno of the Czech Republic. Approximately 1000 patients are hospitalized in the Department of Internal Cardiology Medicine, where recruitment will take place annually. The physicians in the Department have an association with the CR program and are generally supportive of patients referrals.

The institution also has an outpatient CR program. It is a comprehensive program consisting of exercise and education of lifestyle, and it is delivered dominantly by physiotherapists. Indicated patients have to complete cardiopulmonary exercise test before CR enrollment and post-program. There is practically no waiting list, and patients commence CR approximately 4 to 12 weeks post-discharge, depending on their clinical status and preferences. It involves 3 sessions a week for 2 months (total 24 trainings). The health insurance company completely covers the CR program. There could be a problem with parking because of a few places.

### 2.3 Translation and cross-cultural adaptation

We aimed to follow best practices to ensure the translation was accurate, high-quality, and culturally-relevant.^[17–20]^ The following steps were undertaken: preparation, translation, reconciliation/harmonization, and cultural adaptation, piloting, and finalization. For preparation, permission was obtained from Prof Grace to translate the English-language scale, needed experts were invited to the project (professional translator, CR experts), and the target audience was finalized (patients of UH Brno, Czech Republic, indicated for phase II/outpatient CR). In the second step, the source version was translated by a professional translator whose native language was Czech. Next, 2 bilingual CR experts reviewed the translation, considered any conceptual discrepancies with the English version, and identified any wording that might not be culturally applicable. The revised version was then piloted in 50 target patients who had not been to CR as well as CR participants using a think-aloud protocol and follow-up semi-structured interview regarding clarity, relevance, and omissions. The expert team considered all the information from the pilot and finalized the CRBS-CZE.

### 2.4 Psychometric validation

**Figure 1:**
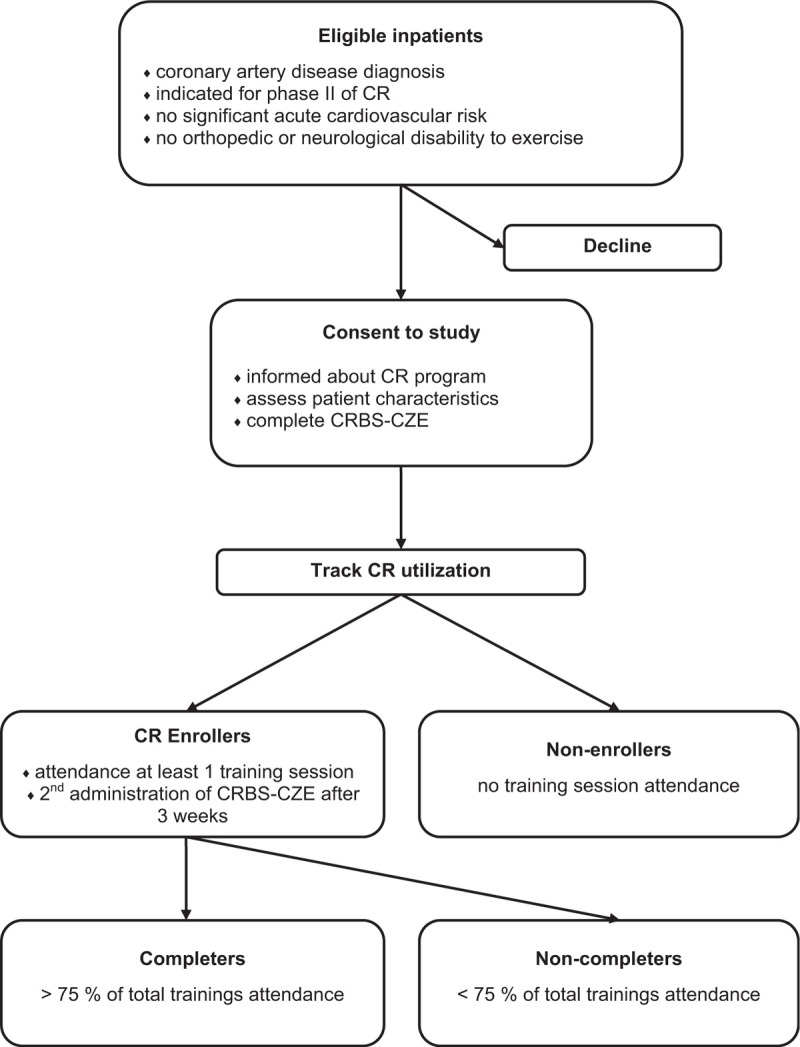
Flowchart of the study protocol.

Eligible patients in the Department of Internal Cardiology Medicine will be approached from January 2020 for 1 year. Consenting patients will be informed about CR and referred. A physiotherapist will provide a questionnaire to the participants assessing their characteristics as well as the CRBS-CZE and will assist them in completion as needed. Thus all data will be secured via self-report, interview with patients, measurement, and will also be extracted from charts where available. Patients’ enrollment, session attendance, and completion of the program will be tracked in the CR charts (this too will be extracted for the study 2 months later). At 3 weeks after enrollment participated, patients will complete CRBS-CZE for the second time. A flowchart of the study protocol is shown in Figure 1.

### 2.5 Participants

Adult coronary artery disease (CAD) patients meeting the criteria for phase II of CR will be recruited (primo manifestation of CAD, acute coronary syndrome, angina pectoris, percutaneous coronary intervention, coronary artery bypass graft, heart failure, arrhythmia, valve defect). Exclusion criteria were as follows: no significant acute cardiovascular risk, no implanted cardioverter-defibrillator or pacemaker, no orthopedic or neurological disability impeding ability to exercise, no mental condition precluding participation (schizophrenia, advanced dementia).

The rule of thumb for factor analysis is 10 participants per variable (at least 5), which would be 210 patients. The expected number of research group patients is 300 to 600 respondents. This is also a reasonable sample size to establish the validity of the translation, as demonstrated in previous CRBS translations.^[21,22]^

### 2.6 Sample characteristics

The sociodemographic and clinical characteristics of the participant will be obtained. Some of these variables were also used to test the content validity of the CRBS-CZE, given many represent or are related to CR barriers or groups that are less likely to participate. Sociodemographic characteristics will include age, sex, marital status, highest educational attainment, income, work status (including shift work, degree of occupational exertion for those employed). Clinical characteristics will include diagnosis, risk factors (anthropometrics, blood pressure, lipids, diabetes), heart-healthy behaviors (physical activity, mins of moderate-vigorous intensity activity/week; tobacco use; harmful use of alcohol, number of standard drinks), and psychosocial well-being (stress, visual analog scale, 1–10; depression/anxiety, diagnosed or subjective). Also, the time to travel to the center will be assessed.

### 2.7 CRBS

This scale evaluates patients’ perception of the degree to which patient, provider, and health system-level barriers affect their CR enrollment and participation (all items applicable to enrollers and non-enrollers alike). The English version consists of 21 items (barriers), related to 4 subscales – healthcare system factors, logistical barriers, work/time conflicts, comorbidities/functional status (although some translations consist of 5 subscales). These items are rated on a 5-point Likert scale (1 – strongly disagree, 5 – strongly agree). Higher scores indicate greater barriers to CR. There is also an open-ended item at the bottom where patients are asked to report any additional factors. Criterion validity has been established in the English version and many of the translations, demonstrated by significant differences in CRBS scores by CR use.^[21,23]^

Where participants completed >80% of the items, a mean total score will be computed. Subscale scores will also be computed based on the results of the factor analysis.

### 2.8 Outcomes for validating the CRBS-CZE and CR utilization

Patients attending at least 1 training session will be considered enrollers by 12 weeks post-discharge. The rest of them will be qualified as non-enrollers. Patients attending >75% of total training will be marked as completers and <75% of total attendance as non-completers. This will be used to test criterion validity. As outlined above, differences in barrier scores by some patient characteristics will also be assessed to investigate content validity; these will include sex, age, travel time, indication (primo manifestation of CAD vs chronic problems), socioeconomic indicators, and risk factor burden.

### 2.9 Analysis

The process of reconciliation/harmonization and cultural adaptation, piloting, and finalization will be documented in detail, and any associated revisions to items specified. For the psychometric validation, Statistica TIBCO (Software Inc, Palo Alto, USA) will be used, and *P* < .05 will denote statistical significance. First, data to characterize the sample will be processed via descriptive statistics – mean, standard deviation, as well as frequencies and percentages. We will consider the completeness of the scale by using content analysis to code any additional barriers reported by respondents. We will assess factor structure with maximum likelihood factor analysis with oblique rotation. Factors with Eigen values >1 will be extracted according to the Kaiser–Guttman criterion. The scree plot will also be considered to confirm the appropriate number of factors. Factor loadings >3 will be interpreted in terms of subscale determinations. To evaluate the internal reliability of CRBS-CZE, we will use Cronbach alpha. To compare barriers by utilization indicators to test criterion validity, we will use a *t* test and Pearson correlation, as applicable. To test construct validity, differences in CRBS total scores will be compared by the sociodemographic and clinical characteristics outlined above, using the same 2 tests as applicable. Finally, interpretation of highest subscale and item scores will ensure to identify key barriers in this setting.

### 2.10 Results of translation and cross-cultural validation of CRBS

The translation was completed in 2019 and is shown in Table 1. In the phase of reconciliation/harmonization and cultural adaptation, revisions in wording were made to the following items: 1 to 7, 10 to 11. Some example additions were made to specify these items, and for clarification, we added point 0 (not applicable) to the Likert scale. In the pilot phase, the patients reported the questions were generally clear, relevant, and comprehensive. There were no additional barriers raised, among non-enrollers or enrollers alike. Some other revisions in wording were made to the following items: 3, 7, and 16.

**Table 1:**
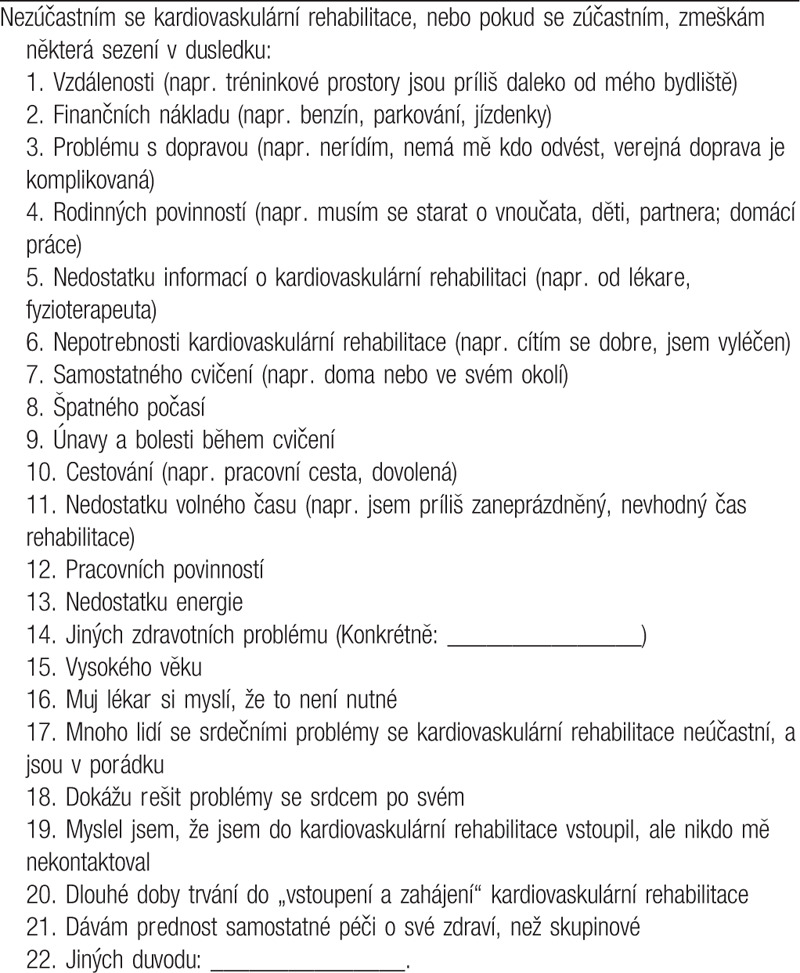
CRBS-CZE.

## 3 Discussion

CR participation is associated with reductions of recurrent cardiovascular events and mortality, decreased hospitalization, and improvements in quality of life.^[6,24,25]^ Despite these benefits and recommendations to refer patients, utilization of CR is low.^[26–28]^ Older people, women, patients with comorbidities, unemployed and single persons, less educated people, those with lower income, and those that live farther from CR facilities have even lower enrollment and adherence to center-based training programs, despite in many cases, greater need.^[28,29]^ Studies demonstrating the effectiveness of CR in the Czech Republic already exist, even in Brno, but there are no studies about utilization and barriers to CR. Given the burden of CVDs, this work will fill this critical gap.^[30]^

Low utilization of CR is a global problem. Thus, strategies to improve utilization of rehabilitation programs have been developed and tested and now demonstrated rigorously to work in high-resource settings.^[31,32]^ Once the specific, critical barriers in this setting are identified, we will undertake more research to determine which of these strategies might be most relevant and effective here. We will conduct further research to establish specific, implementable materials and protocols to overcome the barriers and test them to ensure there is an evidence base to confirm effectiveness. We assume that one of the most critical barriers to CR in the Czech Republic will be the long commuting time to training facilities. One alternative is the use of telemedicine technology. CR delivered using this technology appears to be at least as effective as standard training programs in improving modifiable cardiovascular risk factors and functional capacity, and could enhance utilization by providing additional options for patients who cannot attend center-based training.^[32,33]^ Indeed, we currently have a trial to test such a model in our setting, which, if proven effective, could also be applied to overcome utilization barriers for our population.^[34]^

In conclusion, through this study, we hope to understand the barriers in our region better, so we can ultimately address them to improve access to secondary prevention care for CVDs. While this is a single-center study, given the similarity of the setting to other centers in East-Central Europe, we hope the results of this work can inform efforts to improve CR access across the region.

## Acknowledgments

The authors are grateful to Dr Gabriela Ghisi for sharing her expertise regarding best practices in translation and cross-cultural adaptation of patient materials and measures. The authors also want to thank Dr Katerina Batalikova for the professional translation of the CRBS.

## Author contributions

**Conceptualization:** Ladislav Batalik.

**Cross-cultural validation:** Petr Winnige, Ladislav Batalik.

**Data collecting:** Petr Winnige, Katerina Filakova, Jakub Hnatiak.

**Methodology:** Petr Winnige, Ladislav Batalik, Sherry L Grace.

**Project administration:** Dosbaba Filip, Ladislav Batalik, Katerina Filakova, Jakub Hnatiak.

**Supervision:** Sherry L Grace, Dosbaba Filip.

**Writing – original draft:** Petr Winnige.

**Writing – review and editing:** Sherry L Grace.

Ladislav Batalik orcid: 0000-0003-2147-1541.
